# The role played by perivascular cells in kidney interstitial injury 

**DOI:** 10.5414/CN107371

**Published:** 2012-03-05

**Authors:** Andres Rojas, Fan-Chi Chang, Shuei-Liong Lin, Jeremy S. Duffield

**Affiliations:** 1 Renal Division & Center for Lung Biology, Department of Medicine, and Institute of Stem Cell & Regenerative Medicine, University of Washington, Seattle, WA, USA, and; 2 Department of Internal Medicine, National Taiwan University Hospital, Taiwan, South Korea

**Keywords:** pericytes, kidney fibrosis, capillaries

## Abstract

Fibrosis of the kidney is a disease affecting millions worldwide and is a harbinger of progressive loss of organ function resulting in organ failure. Recent findings suggest that understanding mechanisms of development and progression of fibrosis will lead to new therapies urgently required to counteract loss of organ function. Recently, little-known cells that line the kidney microvasculature, known as pericytes, were identified as the precursor cells which become the scar-forming myofibroblasts. Kidney pericytes are extensively branched cells located in the wall of capillaries, embedded within the microvascular basement membrane, and incompletely envelope endothelial cells with which they establish focal contacts. In response to kidney injuries, pericytes detach from endothelial cells and migrate into the interstitial space where they undergo a transition into myofibroblasts. Detachment leads to fibrosis but also leaves an unstable endothelium, prone to rarefaction. Endothelial-pericyte crosstalk at the vascular endothelial growth factor receptors and platelet derived growth factor receptors in response to injury have been identified as major new targets for therapeutic intervention.

## Introduction 

Renal fibrosis is the final common manifestation of a wide variety of chronic kidney diseases (CKD). Fibrosis is considered a maladaptive repair process and its hallmarks include chronic inflammation and a persistent injury state that promotes the expression of growth factors, fibrogenic cytokines and proteolytic enzymes [1, 2]. In fibrosis, there is an intense recruitment and proliferation of myofibroblasts in the interstitial space, which synthesize and deposit components of pathological, fibrotic extracellular matrix that becomes scar tissue, leading to progressive remodeling and destruction of normal kidney tissue architecture. The damage of the kidney is so severe in some cases that dialysis or transplant of the kidney is required. Fibrosis of the kidney is almost always associated with cardinal features: inflammation (leukocytes), tubule injury/atrophy and peritubular capillary rarefaction [2, 3, 4, 5, 6]. In addition there are frequently changes in the glomeruli which mirror those in the remainder of the kidney: glomerulosclerosis, mesangial expansion, capillary loop loss, inflammation. Although it has been questioned whether fibrosis in the kidney is secondary to nephron dysfunction rather than intrinsic to kidney disease progression, increasing evidence points to fibrogenesis as central to dysfunction and attrition of the nephron. Targeting fibrosis will therefore prevent nephron demise. 

**Figure 1.**
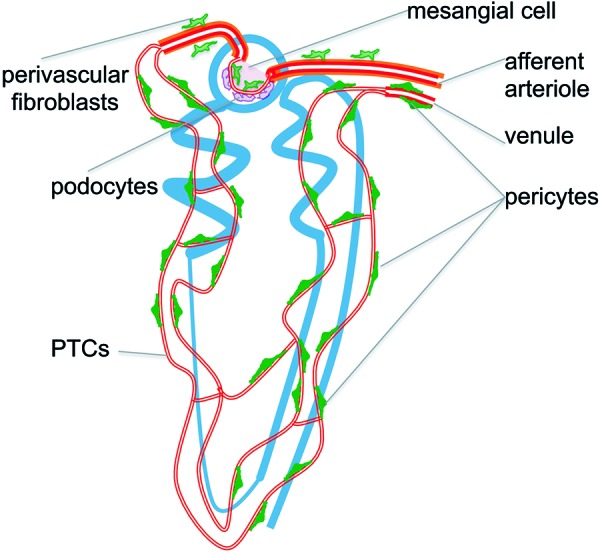
A schema showing the relationship between the nephron and the microvasculature of the nephron and showing the attachment of pericytes to capillaries and the close association of perivascular fibroblasts to the arterioles.

Taking a novel genetic approach to study mechanisms of fibrosis, recent investigations have identified the myofibroblast precursor as a discrete cell type known as “pericyte” that is relatively abundant in the adult kidney, predominantly embedded in the peritubular capillaries where they play active roles in microvascular health (Figure 1) [2, 3, 7, 8]. This review will inform about pericytes drawing on studies in the kidney and other organs, and show that signaling between pericytes and endothelial cells is central to the development of not only fibrosis but also capillary rarefaction and inflammation. 

## Pericytes – mural cells of capillaries that play roles in angiogenesis and vessel stability 

**Figure 2.**
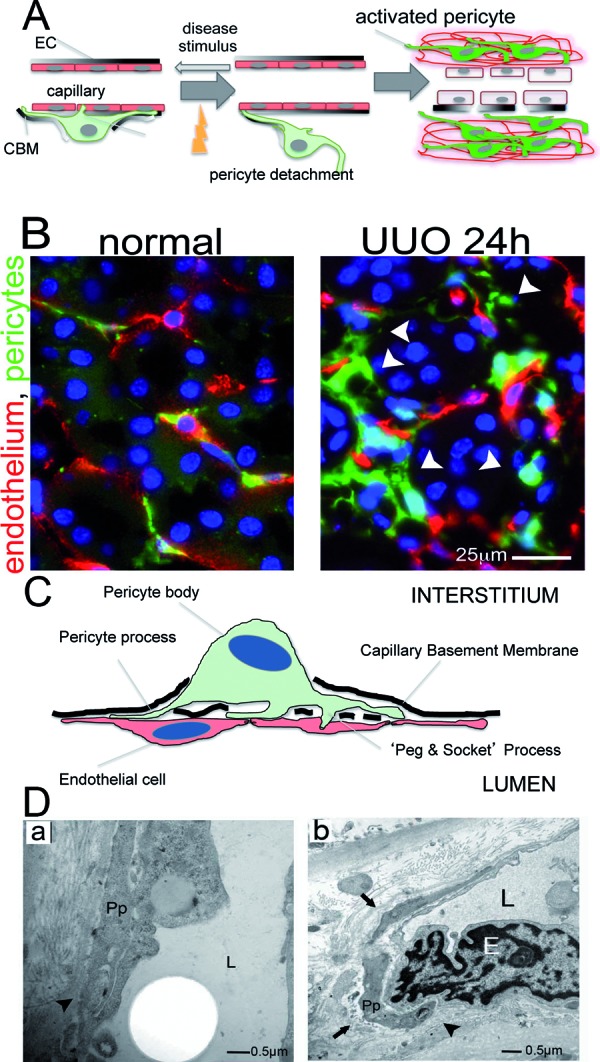
Kidney pericytes and their response to injury. (A,B) Schematic (A) and fluorescence images (B) from *Coll-GFP* mice of pericytes (PCs, green) and capillary endothelial cells (ECs, immunostained for CD31 as red) in normal kidney and 24 hours after unilateral ureteral obstruction (UUO). In response to kidney injury, PCs detach themselves from ECs, spread, migrate (arrowheads in B) and increase collagen expression (become more green). Progression of this process (A) leads to unstable vasculature, capillary loss and interstitial matrix expansion. (C,D) Schematic (C) and electron microscopy images (human sample) (D) of PC-interaction with EC in normal kidney. PC processes are enveloped in capillary basement membrane (CBM) (arrows) where intimate connections and cell : cell signaling occurs known as “peg and socket” junctions (arrowheads). L = capillary lumen, E = EC, Pp = pericyte process.

Pericytes are active cells that have an intimate communication with EC by direct physical contact in a number of cytoplasmic regions including specialized invaginations of cytoplasm called “peg and socket” where paracrine signaling is believed to occur (Figure 2A) [3, 9] that play key roles in angiogenesis, vessel maturation, remodeling and maintenance of the microvasculature via the secretion of growth factors or modulation of the extracellular matrix [9, 10]. The coverage of EC by pericytes varies considerably from different types of microvascular beds within an organ, (observed in lung carcinoma [11]), as well as the density of capillary coverage from organ to organ throughout the body, being the highest in capillaries of the retina and brain [3, 7, 12, 13]. 

Pericytes perform contractile functions in capillaries, regulating their permeability [14]. For example, functional studies have demonstrated that the expression of pericyte contractile microfilaments (actin, myosin) and intermediate filaments (desmin, vimentin) are necessary to control capillary diameter in whole retina and cerebellar slices [15, 16]. It has also been established that when capillaries lose pericytes, they become unstable, tending toward excessive dysregulated angiogenesis, hemorrhage, dilatation and aneurysm formation, and also rarefaction. Collectively these microvascular changes lead to generalized conditions such as edema, tissue ischemia, specific vascular conditions that mimic diabetic retinopathy, and even embryonic lethality due to hemorrhage [9, 17]. Pericytes can be distinguished from perivascular fibroblasts (also known as fibrocytes or adventitial cells) which surround arterioles and have no connection with endothelium (Figure 1). They can also be distinguished from vascular smooth muscle cells (VSMCs), which surround larger blood vessels, by their location, since VSMC are separated from endothelial cells by an internal elastic lamina (Figure 1) and do not completely share molecular (Table 1) marker expression [3]. 

The vascular sprouting (angiogenesis) that leads to fully formed vasculature is mediated by pericyte-endothelial interactions, and is regulated by multiple cell: cell signaling pathways [9, 18, 19, 20]. In embryonic development, the secretion of platelet-derived growth factor PDGF-BB by tip ECs, binds in combination with heparan sulfate, to its receptor PDFGR-β located on pericytes. This ligand–receptor binding triggers pericyte proliferation and migration along the newly formed vascular sprout which stabilizes the newly formed vessel [21, 22, 23]. Other pathways known to be involved in endothelial-pericyte crosstalk include angiopoietin/Tie2 [24, 25] transforming growth factor (TGF) superfamily signaling [26, 27] and vascular endothelial growth factor (VEGF) [18, 28]. 

In the brain, where pericytes were first described in 1873 by the French scientist Charles-Marie Benjamin Rouget [7, 28] they are necessary for the integrity of the blood-brain barrier. Firstly, in combination with ECs they maintain the capillary basement membrane which helps to maintain the homeostasis of the tissue and promote cell survival [14, 29]. In addition, two groups, independently, showed that during development pericytes regulate the formation of tight junctions and its absence in the brain allows transcytosis in brain capillary ECs [30]. In these specialized capillaries, an absolute pericyte coverage (ratio of EC: pericytes is 1 : 1) is essential for EC integrity and maintenance of the blood-brain barrier [29, 30, 31, 32]. 

Supporting the role of pericytes in development of the microvasculature, recent studies using a 3-dimensional collagen vessel formation assay, show that both ECs and pericytes are highly migratory during initial tube assembly and during the ensuing vessel-maturation events. In these assays, in the absence of ECs, pericytes did not migrate in a directed manner. In contrast, when ECs were present in the 3-D assay with pericytes, pericyte motility was markedly induced and this was directional motility toward the endothelium. Without ECs, pericytes displayed significantly reduced velocity, and the cells tended to move in a random way [33]. In addition to EC and pericytes cross signaling during vessel formation, the formation of capillary basement membrane during capillary development in vivo correlates with the arrival of pericytes to forming EC tubes. The formation of this vascular basement membrane allows for increased tube stabilization and has been shown to restrict EC vessel diameter [33, 34, 35]. 

Cancer growth is dependent on angiogenesis, and cancers recruit neovessels, including pericytes. In gastric cancer malignant epithelial cells which generate PDGF-B results in increases of PDGFR-β expression in the tumor and higher pericyte coverage around vessels, thereby promoting the growth of the tumor [36]. In recent studies of pancreatic cancer, pharmacological inhibition (XL880) of vascular endothelial growth factor (VEGF)-receptor 2 (VEGFR2) resulted in a decrease of capillary basement membrane formation and there was a 71% reduction in pericyte coverage. The absence of new pericyte coverage of the neovessels prevented stabilization and formation of new vasculature promoting hypoxia and apoptotic cells death in cancer cells [37, 38]. 

## Kidney pericytes are myofibroblast precursors 

Although pericytes were described in the kidney nearly 30 years ago [2, 39] they have been little studied until recently. Using a transgenic mouse that expresses green fluorescent protein (GFP) under the regulation of the Collagen Type I promoter (Coll1a1), numerous cells in kidney cortex and medulla were identified attached by multiple long processes to peritubular capillaries (PTCs) (Figure 1). Closer inspection indicated that these Coll-GFP cells were predominantly within the capillary basement membrane (CBM) and electron microscopy identified numerous cell bodies or cell processes sheathed in CBM normal kidney cortical peritubular capillaries (PTCs) (Figure 2A, B, C, D) [3, 6, 40]. These processes exhibited pedicle-like attachment plaques to ECs and invaginations of both EC membrane with process membrane to form “peg and socket” processes. These features are all characteristics of mesenchyme derived cells known as pericytes [2, 6, 41]. (Figure 2C, D). In addition to pericytes a population of perivascular fibroblasts (also known as fibrocytes or adventitial cells) surrounding arterioles was identified by expression of the Coll-GFP transgene (Figure 1). 

Pericytes in the normal kidney have been studied for expression of markers (Table 1) and consistently express PDGFR-α and PDGFR-β. In post-natal kidney the pericytes express αSMA and NG2 but in adult kidneys these markers are not downregulated (Table 1). 

Injury responses in the adult kidney were studied, and pericytes were seen to detach, upregulate collagen expression, migrate away from the endothelium and reactivate the pericyte marker NG2 (Figure 2A, B) [6]. Four days after injury the population of Coll-GFP expressing pericyte cells, now in the interstitium, had expanded markedly and the vast majority activated expression of the myofibroblast marker αSMA. Careful mathematical kinetic analysis of the expansion of Coll-GFP pericytes with time after injury strongly suggested that the appearance of Coll-GFP expressing myofibroblasts could be explained by detachment, migration and expansion of the original population of pericyte cells (Figure 2B) [6]. 

**Figure 3.**
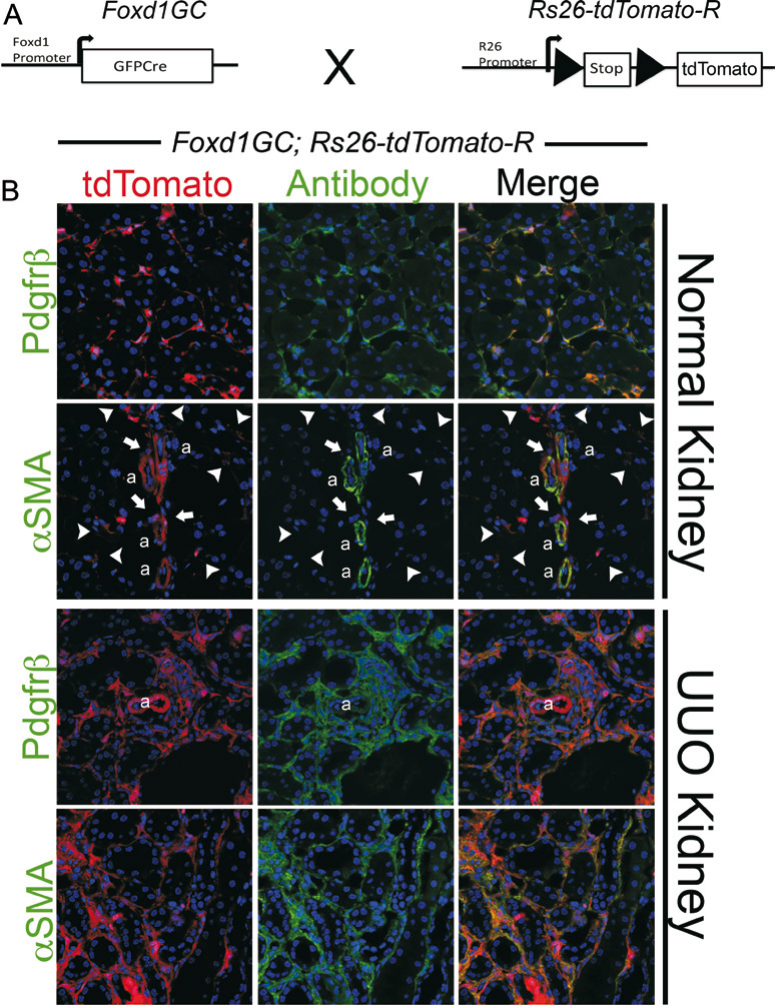
Results of fate mapping of Foxd1 progenitors in adult and injured kidney using the *Foxd1-Cre;Rosa26-tdTomatoR* mouse. A: Schema showing the cross of *Foxd1-Cre* recombinase allele with *TdTomato* reporter allele driven by the universal promoters at the *Rosa26* locus. Bigenic mice recombine genomic DNA at the Rosa locus only in cells that have activated *Foxd1* gene in nephrogenesis. B: Confocal images of kidney cortex showing in normal adult kidney large numbers of perivascular cells, which all co-express PDGFR-β. VSMCs of the kidney arterioles are also derived from *Foxd1*-progenitors and co-express αSMA intermediate filament, but none of *Foxd1*-derived pericytes (arrowheads) or perivascular fibroblasts (arrows) express αSMA. In kidney injury (shown here is UUO Day 7) the pericyte and perivascular fibroblast populations expand and continue to express PDGFR-β. However, now all of the expanded population of interstitial Foxd1-progenitor derived cells co-express αSMA, the marker which defines these cells as myofibroblasts.

In order to study the fate of pericytes in kidney injury further, a genetic fate mapping approach was taken. Mice expressing the DNA recombinase enzyme Cre at the loci of transcription factors expressed by discrete subpopulations embryonic kidney precursors (stem cells) in metanephric mesenchyme were generated [2]. The Foxd1 transcription factor gene locus was selected since Foxd1 progenitors become kidney pericytes as well as mesangial cells and vascular smooth muscle [2, 7, 42, 43, 44]. By expressing the Cre transgene under regulation of the Foxd1 locus in Rosa26 reporter mice all pericytes, vascular smooth muscle and mesangial cells of the kidney were labeled (Figure 3) [2]. In response to kidney injury (ischemia reperfusion injury or ureteral obstruction) over 2 – 3 weeks there was a 15-fold increase in the Foxd1 reporter-labeled progeny and these cells all activated the myofibroblast marker αSMA (Figure 3) [2], identical to the fate of Coll-GFP cells in the *Coll-GFP* mouse [6]. These findings strongly suggested that pericytes were the predominant if not only source of myofibroblasts in mouse kidney injury. The fate mapping findings were further supported by cohort labeling using the conditional, tamoxifen sensitive, CreER recombinase at the Foxd1 locus [2]. In these studies a cohort of only 20% of Foxd1 progenitor-derived pericytes was labeled by tamoxifen exposure during nephrogenesis. Adult kidneys were injured and the labeled cohort expanded 15 fold, activated αSMA and represented 20% of the total myofibroblast population in the injured kidney. Together, these powerful genetic studies indicate that pericytes are the major progenitor of myofibroblasts in the kidney models we study. In the same studies all the epithelia of the kidney were fate mapped by a similar approach, but none of the mapped epithelia gave rise to myofibroblasts [2]. Subsequent to these studies four other groups have independently fate mapped epithelial of the kidney in three different disease models and none of them identify kidney epithelium as a source of myofibroblasts [2, 42, 45, 46]. In separate studies the fate of leukocytes as progenitors of myofibroblasts was studied by bone marrow chimerism techniques, but no leukocytes were identified as myofibroblast progenitor [7, 8, 47]. These studies therefore point to pericytes as the major if not only progenitor of myofibroblasts in kidney injury. 

## Endothelial-pericyte crosstalk – the role of dysangiogenesis in fibrogenesis 

The fact that pericytes detach, migrate and transition into myofibroblasts after kidney injury and the fact that pericytes form direct communications with endothelial cells at peg and socket junctions, have promoted new investigations aimed at establishing and dissecting the key cell-signaling pathways regulating the initiation and progression of renal fibrosis. Two of these are VEGF receptor and PDGF receptor cell-to-cell signaling pathways. 

**Figure 4.**
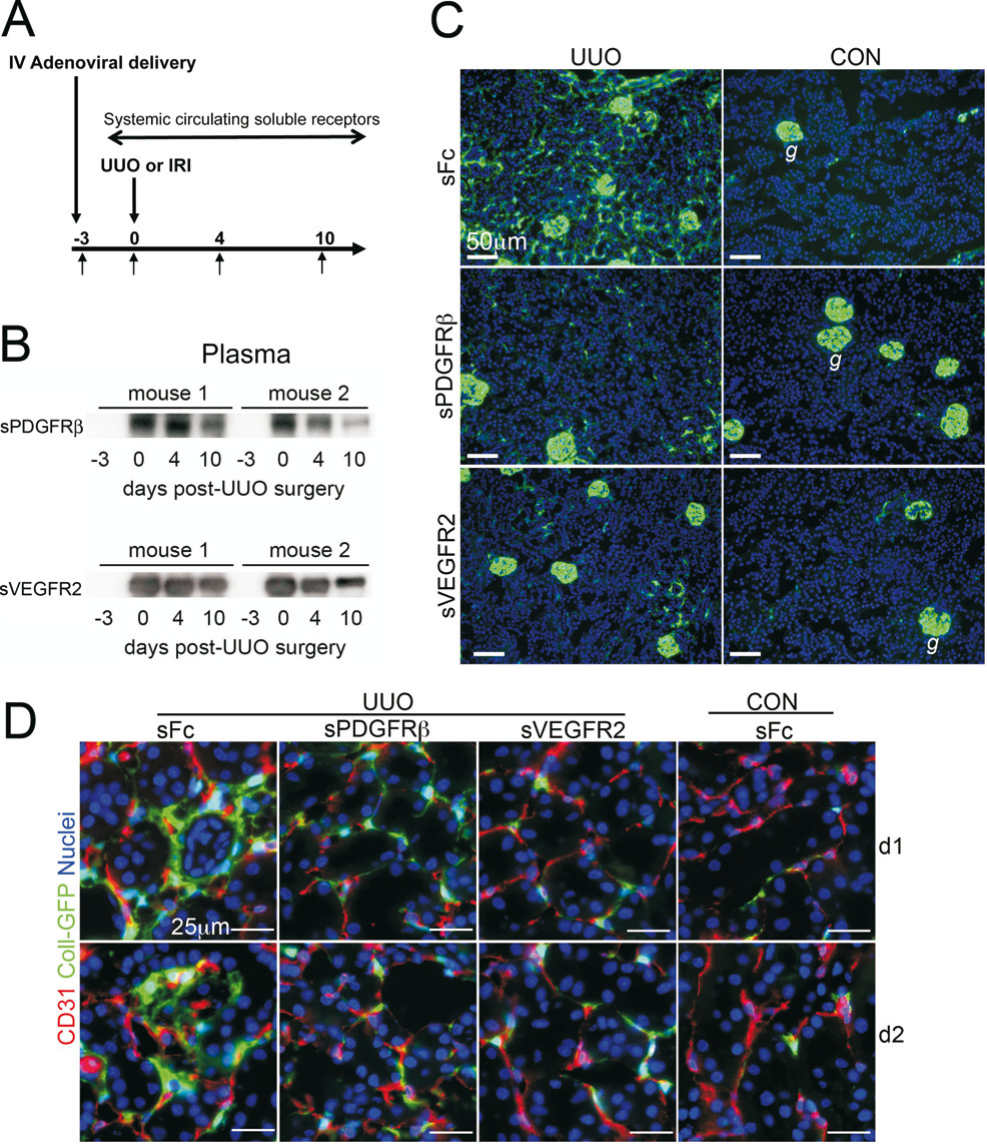
Pericyte detachment, transition to the myofibroblast phenotype and interstitial fibrosis can be blocked by inhibiting the activation of VEGFR2 and PDGFR-β with circulating soluble receptor ectodomains: A: Schema showing the experimental protocol. Using engineered adenoviruses as gene delivery tools, the liver, which is the main target organ for IV delivery of adenoviruses, synthesizes and releases into the circulation high levels of soluble receptors for the duration of the experiments. Three days after viral delivery kidney injuries were performed and analyzed up to 10 days later. B: Western blots detecting sPDGFR-β or sVEGFR2 in 1 µl of mouse plasma. C: Low power immunofluorescence images of kidneys Day 4 after UUO or sham surgery (CON) in *Coll-GFP* reporter mice that received control Fc-virus or viruses producing sPDGFR-β or sVEGFR2. Note that activated Coll-GFP+ pericytes/myofibroblasts are much more abundant in the interstitium of control (sFc) diseased kidneys compared with kidneys that were exposed to sVEGFR2 or sPDGFR-β (g = glomerulus where podocytes also can be seen). D: High power fluorescence images of pericytes (green) and endothelial cells (red) in kidneys on Day 1 or 2 after UUO injury or sham surgery (CON). Note that in normal kidney pericytes are attached to endothelial cells but 24 h after UUO in kidney exposed to sFc (control virus) there is detachment spreading and migration of pericytes from the endothelium. In the presence of either sPDGFR-β or sVEGFR2 this detachment is almost completely prevented. Similar findings persist 2 days after UUO injury.

One of the first reports on this matter, was made by Lin and colleagues [18]. Using viruses as gene delivery tools, the investigators delivered soluble ectodomain of VEGFR2 to block the kidney endothelial-restricted VEGFR2. In separate studies they delivered soluble ectodomain of PDGFR-β to block signaling at kidney PDGFR-β which is restricted to kidney pericytes (Figure 3) (Table 1). Either of these soluble blocking receptors profoundly inhibits the triad of microvascular rarefaction, interstitial fibrosis, and inflammation in animal models of non-glomerular kidney injury (Figure 4) [18]. Moreover, blocking either the endothelial receptor (VEGFR2) or the pericyte receptor (PDGFR-β) alone almost completely prevented pericyte detachment from the peritubular capillaries of the kidney (Figure 4), providing persuasive evidence that pericyte detachment is central to many of the manifestations of chronic kidney diseases not just the development of fibrosis. Since VEGFA is generated by pericytes and they are attached to endothelial cells where VEGFR2 is located, it is likely that pericytes in the kidney regulate signaling at the endothelial VEGFR2. The investigators discovered that as pericytes detach and migrate from ECs in response to injury, they rapidly switch VEGFA isoforms. The normal VEGFA is VEGF164, but activated pericytes generate VEGF120 and VEGF188, both are known to signal differently at VEGFR2 and promote unstable angiogenesis [18]. 

The PDGFR-β is completely restricted in its expression to pericytes, vascular smooth muscle and the mesangial cells in the kidney [2, 3, 6, 23]. The PDGFs that bind and activate the receptor are generated by a number of cells in kidney injury, but the endothelial cells, which are directly attached to pericytes, are a major source of new PDGF-BB in the injured kidney. It is very likely therefore that endothelial signaling to pericytes is also important in the pericyte detachment, migration and the development of fibrosis. 

In follow-up studies, blockade of either PDGFR-β or PDGFR-α (which is also restricted in expression to kidney pericytes, mesangial cells and vascular smooth muscle of kidney arterioles) in two rodent models of non-glomerular kidney disease using antibodies effective in humans [18, 48], also blocked pericyte detachment, and the subsequent triad of inflammation, fibrosis and microvascular rarefaction. These antibodies showed highly similar efficacy to the soluble PDGFR-β receptor studies and reiterate that targeting single receptors restricted to pericytes is sufficient to prevent fibrosis, inflammation, and rarefaction of the microvasculature. 

These studies identify dysregulation of signaling pathways that normally regulate angiogenesis as central to the development of kidney fibrosis and chronic kidney disease. Moreover they place the vital crosstalk between endothelial cells and pericytes at the center of injury responses in the kidney, regardless of epithelial events. Furthermore, they add new meaning to fibrosis, because for the first time, targeting fibrogenesis will have far greater impact than simply limiting interstitial matrix. 

## Conclusions 

By taking a fresh look at the development of kidney fibrosis using modern genetic tools, new studies have identified Foxd1 progenitor-derived pericytes and perivascular fibroblasts, but not epithelial cells, as the precursor cells of scar-forming myofibroblasts. These results were supported by the use of animal models in which using two different kidney injuries. It has been established that pericytes detach, migrate and transition into cells that produce high levels of collagen and other pathological matrix proteins. Detachment of pericytes from the microvasculature leaves the microvessels unstable and prone to dysangiogenesis and rarefaction. 

Inspired by these new discoveries, early candidate therapies have been identified in models of acute and chronic kidney injury that target either the endothelial VEGFR2 or the pericyte PDGFR-α or PDGFR-β. 

Further studies of kidney pericytes are urgently warranted to understand: a) the molecular mechanisms of pericyte attachment and detachment from vessels in response to injury; b) determine whether a myofibroblast can transition again to pericyte functions (tissue regeneration); c) understand the role of the pericyte in development and homoeostasis; d) define the molecular mechanisms by which injured epithelial cells signal to perivascular cells 

## Funding support 

The Duffield Lab is funded by NIH Grants DK73299, DK84077, DK8739, Genzyme Research In Progress, University of Washington, Institute for Stem Cell & Regenerative Medicine, Nephcure Foundation, and a Research agreement from Regulus therapeutics. 

**Table 1.** Proportion of Coll1a1-GFP+ pericytes that co-express the following markers in normal rodent kidney.

Marker	P12 days	P16 days	12 weeks
NG2	98.4%	74%	3%
PDGFR-β	100%	100%	100%
PDGFR-α	100%	100%	100%
CD73	100%	100%	100%
αSMA	100%	94%	0%
CD45	0%	0%	0%
